# Bone Composition Changes and Calcium Metabolism in Obese Adolescents and Young Adults Undergoing Sleeve Gastrectomy: A Systematic Review

**DOI:** 10.3390/jcm14020393

**Published:** 2025-01-10

**Authors:** Viorel Dejeu, Paula Dejeu, Anita Muresan, Paula Bradea, Danut Dejeu

**Affiliations:** 1 Bariatric Surgery Department, Life Memorial Hospital, Calea Grivitei 365, 010719 Bucuresti, Romania; office@doctordejeu.ro; 2 Laboratory Medicine Unit, Betania Medical Center, Menumorut 12, 410004 Oradea, Romania; 3 Surgical Oncology Department, Emergency County Hospital Oradea, Strada Gheorghe Doja 65, 410169 Oradea, Romania; amuresan@uoradea.ro (A.M.); ddejeu@uoradea.ro (D.D.); 4 Surgery Department, Faculty of Medicine and Pharmacy, University of Oradea, Piata 1 Decembrie 10, 410073 Oradea, Romania; 5 Gastroenterology Unit, Betania Medical Center, Menumorut 12, 410004 Oradea, Romania; paula.bradea@betania-centrulmedical.ro; 6 Bariatric Surgery Department, Medlife Humanitas Hospital, Strada Frunzisului 75, 400664 Cluj Napoca, Romania

**Keywords:** obesity, bariatric surgery, adolescents

## Abstract

**Background/Objectives**: Sleeve gastrectomy (SG) is increasingly used to treat severe obesity in adolescents, but its effects on bone health during this critical period of bone accrual are not fully understood. This systematic review aims to evaluate the impact of SG on the bone mineral density (BMD), bone microarchitecture, marrow adipose tissue (MAT), and bone turnover markers in adolescents. **Methods**: A comprehensive literature search was conducted to identify studies assessing bone health outcomes in adolescents undergoing SG. Nine studies met the inclusion criteria, comprising prospective cohorts, observational cohorts, and one randomized controlled trial, with sample sizes ranging from 10 to 197 participants aged 13 to 25 years, and a total sample size of 597 individuals. Data were extracted and synthesized into tables summarizing changes in BMD, bone microarchitecture, MAT, and bone turnover markers. **Results**: SG in adolescents is associated with significant reductions in areal BMD at critical skeletal sites, particularly the femoral neck and total hip, with decreases ranging from −4.7% to −8.9%. Studies utilizing high-resolution peripheral quantitative computed tomography (HRpQCT) reported deteriorations in bone microarchitecture, including a decreased trabecular number, increased trabecular separation, and reduced cortical thickness. Two studies observed significant increases in MAT at the lumbar spine post-SG. Elevated bone turnover markers, particularly C-terminal cross-linking telopeptide (CTX), indicate increased bone resorption following SG. **Conclusions**: SG leads to negative effects on bone health in adolescents, including reductions in BMD, deterioration of the bone microarchitecture, increases in MAT, and elevated bone resorption markers. These findings highlight the need for careful monitoring of bone health and the development of strategies to mitigate bone loss in adolescents undergoing SG.

## 1. Introduction

Obesity during adolescence and young adulthood has escalated into a significant global health concern, with increasing prevalence over recent decades [1,2]. Severe obesity during these critical developmental periods is associated with numerous comorbidities, including type 2 diabetes, hypertension, and dyslipidemia, which can persist into adulthood [3,4,5]. Metabolic and bariatric surgery (MBS), particularly sleeve gastrectomy (SG), has emerged as an effective intervention for significant weight loss and improvement of obesity-related comorbidities in adolescents [6,7].

Adolescence is a pivotal period for bone development, during which individuals achieve peak bone mass—a key determinant of long-term skeletal health and fracture risk [8,9,10]. Interventions that adversely affect bone accrual during this time can have lasting consequences, potentially increasing the risk of osteoporosis and fractures later in life [11,12]. Understanding the impact of SG on bone health in adolescents is therefore of paramount importance.

While SG is effective for weight loss and metabolic improvements, concerns have been raised about its potential negative effects on bone health [13,14,15]. Studies in adults have reported reductions in bone mineral density (BMD) and alterations in the bone microarchitecture following bariatric surgery [16]. The mechanisms may include changes in hormonal profiles, nutrient absorption, mechanical unloading due to weight loss, and alterations in marrow adipose tissue (MAT) [17].

Given these considerations, this systematic review aims to synthesize the current evidence on the impact of SG on bone health outcomes—including BMD, bone geometry, microarchitecture, MAT, and bone turnover markers—in obese children and adolescents. This study represents the first systematic review focusing specifically on the changes in bone composition and calcium metabolism in obese adolescents and young adults undergoing sleeve gastrectomy. As obesity continues to be an escalating global issue, particularly affecting younger populations, understanding its impact on critical periods of bone development is increasingly important. By evaluating the available studies, we seek to understand the extent of bone health alterations following SG in this population and identify gaps in knowledge to inform clinical practice and future research.

## 2. Materials and Methods

### 2.1. Eligibility Criteria

This review considered studies based on the following inclusion criteria: (1) participants were children or adolescents with ages ranging between 14 and 25 years, with obesity, who were undergoing sleeve gastrectomy; (2) studies reported outcomes in terms of bone composition changes, bone mineral density (BMD), marrow adipose tissue (MAT), or calcium metabolism; (3) the study designs included randomized controlled trials, observational studies, or cohort studies; (4) articles were published in English. The exclusion criteria were as follows: (1) studies involving adults only; (2) studies not specifically examining bone outcomes post-SG; (3) case reports, reviews, editorials, or conference abstracts without full data.

### 2.2. Search Strategy

A comprehensive search was conducted in the PubMed, Scopus, and Embase databases up to October 2024. The search aimed to identify all relevant studies on SG and bone health outcomes in obese children and adolescents.

The search terms used included combinations of the following keywords: “sleeve gastrectomy”, “bariatric surgery”, “adolescents”, “children”, “obesity”, “bone mineral density”, “marrow adipose tissue”, “calcium metabolism”, “bone microarchitecture”, and “bone composition”. Boolean operators (AND, OR) were utilized to refine the search. The reference lists of relevant articles were also screened to identify additional studies.

### 2.3. Selection Process

Two independent reviewers screened the titles and abstracts of identified studies. Full-text articles were obtained for studies that met the inclusion criteria or if their eligibility was unclear. Discrepancies were resolved through discussion or consultation with a third reviewer. The selection process adhered to the Preferred Reporting Items for Systematic Reviews and Meta-Analyses (PRISMA) guidelines [18] and was registered in the Open Science Framework, with the registration code osf.io/tk8r3.

### 2.4. Data Items

The data collection process involved extracting information using a standardized form to ensure consistency across the studies reviewed. This form captured various details, including the characteristics of each study, such as the authors, publication year, country, and design of the study. Participant details were also noted, including age, sex, sample size, and baseline Body Mass Index (BMI). Information on the surgical procedures, such as the type of surgery performed and the duration of follow-up, was recorded. Key outcomes related to bone health were meticulously gathered, including areal and volumetric bone mineral density (BMD), bone geometry, microarchitecture, marrow adipose tissue (MAT), bone turnover markers, and levels of calcium and vitamin D. The results and conclusions section documented significant findings related to bone health.

### 2.5. Risk of Bias and Quality Assessment

A quality assessment was performed independently by two reviewers using the Newcastle-Ottawa Scale [19]. Studies were rated on a scale of 0 to 9, with higher scores indicating higher quality. The assessment considered aspects such as sample representativeness, ascertainment of exposure/outcome, comparability of cohorts, and adequacy of follow-up.

## 3. Results

### Study Selection and Study Characteristics

**Figure 1:**
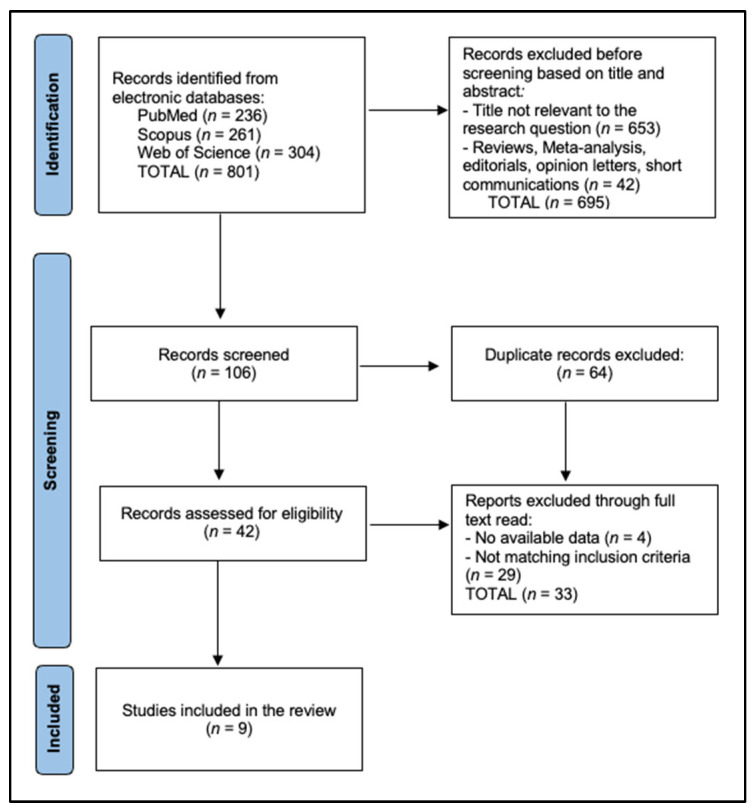
PRISMA flow diagram.

Nine studies were included in the final review [20,21,22,23,24,25,26,27,28], as presented in Figure 1. The studies detailed in Table 1 collectively analyzed data from 592 participants, demonstrating a significant commitment to understanding the effects of various interventions, and were primarily in the United States, with a single exception from Sweden. This breadth of data, gathered through mostly prospective and observational cohort studies, underscores the research community’s effort to derive meaningful insights from a youth demographic, specifically ages 13 to 25. The inclusion of diverse study designs, including the high-quality randomized controlled trial by Järvholm et al. [25], enriches the reliability and depth of the findings.

Across these studies, the variation in sample sizes, from a minimum of 10 in the study by Bredella et al. [20] to a maximum of 197 in the study conducted by Weiner et al. [22], highlights the different scales of research within this field. The quality of the studies, with scores ranging from 7 to 9, reflects rigorous research methodologies and execution. The concentration of research within the USA, apart from one study in Sweden, suggests a robust framework for assessing the long-term outcomes and effectiveness of the interventions explored, although it points to a potential area for expansion in international contexts to validate these findings globally.

The data presented in Table 2 present varied outcomes in terms of the areal bone mineral density (aBMD) following sleeve gastrectomy across multiple studies. Bredella et al. [20] reported a decrease in lumbar spine aBMD from 1.064 to 0.648 g/cm², translating to a −3.7% change, which was not statistically significant. Similarly, Mitchell et al. [24] and Järvholm et al. [25] observed no significant changes in lumbar spine and whole-body BMD, respectively. In contrast, significant reductions in aBMD were noted in the studies by Misra et al. [21,23], with the femoral neck and total hip experiencing notable decreases, −6.9% and −4.7% in one study and −8.9% and −8.4% in another, all with statistically significant *p*-values (*p* = 0.0007 and *p* = 0.0004 for the former; *p* < 0.001 for the latter).

Huber et al. [26,27] observed decreases in the volumetric BMD (vBMD) at the lumbar spine in their respective studies, with changes of −6% and −4.6%, which both achieved statistical significance (*p* < 0.001). Nimmala et al. [28] reported a significant decrease in total hip BMD Z-score, moving from 1.50 to a reduction of −0.75, with a *p*-value of <0.0001. These findings suggest that while some studies observed statistically significant declines in bone density at various anatomical sites post-surgery, others did not, highlighting the variability of sleeve gastrectomy’s impact on bone health.

Table 3 highlights changes in the bone microarchitecture following surgical interventions, as assessed by high-resolution peripheral quantitative computed tomography (HRpQCT). Notably, studies reporting specific microarchitectural changes found statistically significant alterations post-procedure. Misra et al. [21] observed a decrease in the trabecular number and an increase in trabecular separation in the distal tibia, with changes of −8.6% and +10.4%, respectively, which are both significant with a *p*-value of 0.004. Similarly, Misra et al. [23] reported a decrease in cortical thickness and an increase in the buckling ratio at the hip, with measurements of −0.02 ± 0.01 cm and +0.80 ± 0.44, which were both significant with *p*-values of 0.005 and 0.004, respectively.

Mitchell et al. [24] noted a decrease in the trabecular volumetric bone mineral density (vBMD) at the distal radius and tibia, with reductions of −10.4% and −6.1%, respectively, both showing significance (*p* < 0.001). Huber et al. [26,27] reported decreases in vertebral strength at the lumbar spine, with changes of −728 N ± 691 (−6.7%) and −609 N ± 838 (−5.5%), respectively, each with a significant *p*-value of <0.001. Nimmala et al. [28] documented a significant decrease in the trabecular vBMD at the radius, recording a reduction of −7.00 mgHA/cm³, with a *p*-value of 0.002. These findings indicate a general trend of bone structure weakening post-surgery, with significant implications for long-term skeletal health.

Table 4 focuses on the changes in bone turnover markers following sleeve gastrectomy, revealing varying effects, as demonstrated by different studies. Weiner et al. [22] observed significant increases in both osteocalcin and C-terminal telopeptide (CTX), with osteocalcin levels rising by 33.70% (*p* < 0.001) and CTX levels increasing by 57.50% (*p* < 0.001). These substantial elevations indicate a marked increase in bone turnover following surgery, potentially reflecting an acceleration in the bone remodeling processes.

In contrast, Mitchell et al. [24] reported a slight decrease in Procollagen Type 1 N-Terminal Propeptide (P1NP) levels by −5.10%, which was not statistically significant, suggesting a minimal impact on bone formation. However, there was a slight significant increase in CTX levels of 7.00% (*p* = 0.022), indicating a subtle increase in bone resorption. Similarly, Nimmala et al. [28] documented increases in both P1NP and CTX, with P1NP showing an overall increase and CTX levels rising significantly (*p* = 0.0001), suggesting enhanced bone resorption post-surgery. These findings collectively highlight a complex interaction of bone metabolic processes, influenced by sleeve gastrectomy, with implications for bone health that may require monitoring and management in postoperative care.

Moreover, in the studies that reported changes in marrow adipose tissue (MAT) following sleeve gastrectomy, significant increases were observed. Bredella et al. noted a 38.8% increase in MAT at the lumbar spine, with the baseline MAT measurement of 0.27 ± 0.12 rising to 0.36 ± 0.19 post-surgery, showing statistical significance (*p* = 0.016). Similarly, Huber et al. reported a 26.3% increase in lumbar spine MAT, from a baseline of 0.38 ± 0.15 to 0.48 ± 0.19 following the procedure, with this change also proving to be statistically significant (*p* = 0.001).

## 4. Discussion

### 4.1. Summary of Evidence

The findings from this systematic review indicate that SG in obese adolescents is associated with significant reductions in BMD at critical skeletal sites, particularly the femoral neck and total hip. Several studies reported decreases in areal BMD ranging from −4.7% to −8.9%, which were statistically significant [21,23,28]. These sites are essential for supporting an individual’s body weight and are prone to fractures if compromised. The decrease in BMD suggests that SG may negatively impact bone accrual during a critical period of bone mass development, potentially increasing the risk of osteoporosis and fractures later in life.

Alterations in the bone microarchitecture were also observed. Studies utilizing HRpQCT and quantitative CT reported deteriorations in trabecular and cortical bone parameters, including decreases in trabecular number, increases in trabecular separation, and reductions in cortical thickness and vertebral strength [21,23,24,26,27,28]. For example, Uber et al. [26,27] reported a significant decrease in vertebral strength by −728 N ± 691 over 24 months post-SG. These changes reflect a deterioration in bone quality that cannot be captured by BMD measurements alone. The combined effect of reduced bone mass and a compromised microarchitecture raises concerns about the long-term skeletal health of adolescents undergoing SG.

The studies by Bredella et al. [20] and Huber et al. [26] observed significant increases in MAT at the lumbar spine following SG, with increases of 38.8% and 26.3%, respectively. Higher MAT is associated with decreased bone formation and increased resorption, potentially due to shifts in mesenchymal stem cell differentiation favoring adipogenesis over osteoblastogenesis. The positive correlation between weight loss and MAT increase suggests that significant weight reduction may lead to adipogenesis within the bone marrow, exacerbating bone loss.

Elevated bone turnover markers, particularly CTX, indicate increased bone resorption following SG. The studies by Weiner et al. [22] and Nimmala et al. [28] reported significant increases in markers of bone resorption and formation, suggesting an overall increase in bone turnover. For instance, the study by Weiner et al. [22] reported a 57.5% increase in CTX levels at 6 months post-SG. The imbalance between bone resorption and formation suggests that the bone loss may continue unless interventions are implemented to rebalance bone remodeling. The persistence of elevated CTX levels highlights the need for long-term monitoring and management of bone health in this population.

The calcium and vitamin D levels remained within normal ranges post-SG in most studies, with some reporting slight increases due to supplementation. This suggests that the observed bone changes are not solely due to deficiencies in these nutrients. However, adequate calcium and vitamin D intake alone may not be sufficient to counteract the negative effects of SG on bone health, and additional strategies may be necessary.

In a systematic review and meta-analysis by Mitra et al. [29], it was observed that patients who underwent Roux-en-Y gastric bypass (RYGB) or sleeve gastrectomy (SG) exhibited significant decreases in lumbar BMD by −0.96 g/cm² and subtotal body BMD by −0.7 g/cm² and a Z score decline of −1.132, all with a high level of statistical significance (*p* < 0.001). Additionally, bone resorption markers such as C-terminal telopeptide increased by 0.22 ng/mL and osteocalcin by 10.83 ng/mL, indicating heightened bone turnover post-surgery. In contrast, the study by Bezerra et al. [30] also analyzed body composition changes post-bariatric surgery in adolescents, noting significant reductions in fat mass and body weight. Interestingly, this review highlighted that the bone mass did not appear to be adversely affected within the first 12 months post-surgery, presenting a conflicting view compared to the findings of Mitra et al. [29], which can be attributed to the evaluation of multiple bariatric procedures, instead of targeting SG only. Additional differences can be attributed to the age differences.

In the study by de la Cruz-Muñoz et al. [31], the long-term outcomes following metabolic and bariatric surgery (MBS) in adolescents were explored, revealing significant sustained weight loss and remission of comorbid conditions over a decade or more post-surgery. Specifically, participants experienced a mean total body weight decrease of 31.3%, with Roux-en-Y gastric bypass (RYGB) patients showing a 32.0% reduction and laparoscopic adjustable gastric banding (LAGB) participants showing a 22.5% decrease. Comorbidities such as hyperlipidemia, asthma, and diabetes showed a 100% remission rate, with notable reductions also seen in hypertension, sleep apnea, and depression (*p* < 0.001). In a similar manner, the study by Shah et al. [32] assessed the outcomes after bariatric surgery in preteens compared to teens, finding no significant differences in safety or efficacy between the two age groups. Both groups exhibited comparable decreases in BMI over time, with preteens achieving a 16% reduction at 3 months and 20% at 12 months post-surgery.

The study by Ivaska et al. [33] investigated changes in bone metabolism after Roux-en-Y gastric bypass (RYGB) and sleeve gastrectomy (SG), revealing significant increases in bone turnover markers such as CTX, PINP, TRAcP5b, TotalOC, and ucOC, all showing marked elevations postoperatively (*p* < 0.0001 for all). Despite these biochemical changes, no significant alterations were noted in volumetric bone mineral density (vBMD) at the lumbar spine or vertebral bone marrow (VBM) fat six months after surgery. Similarly, the study by Blom-Høgestøl et al. [34] provided insights into bone health a decade after RYGB, with data indicating a high prevalence of osteoporosis (27%) in postmenopausal females or males aged 50 years or older and a significant fraction (8%) of premenopausal females or younger males showing aBMD values below the expected range for their age. Secondary hyperparathyroidism was observed in 31% of the participants, vitamin D deficiency in 33%, and insufficiency in 75%. Additionally, bone resorption markers such as CTX-1 and PINP were notably higher in participants with lower aBMD scores.

The study by Aaseth and Alexander [35] focused on the heightened risk of developing postoperative osteoporosis following bariatric surgery, particularly after Roux-en-Y gastric bypass (RYGB) and sleeve gastrectomy (SG). Their narrative review highlighted that despite the pre-emptive supplementation of calcium and vitamin D, osteoporosis might still manifest, likely due to deficiencies in other critical micronutrients like vitamin K and zinc, which are crucial for bone health. They emphasized that SG and RYGB affect nutrient absorption differently, with SG impacting the absorption of vitamin B12 and D, and RYGB affecting a broader range of nutrients including fat-soluble vitamins. In a similar manner, the study by de Holanda et al. [36] explored the prevalence of morphometric vertebral fractures (MVFs) and its association with bone mineral density (BMD) and biomarkers in patients post-BS. They found a significant prevalence of MVF (17.5%) and noted that the RYGB group exhibited lower BMD values at various sites compared to the SG group, alongside higher rates of secondary hyperparathyroidism and bone turnover markers such as CTX and alkaline phosphatase. Both studies underscore the complex interplay between nutrient absorption, bone health, and the surgical method, stressing the need for thorough postoperative monitoring and individualized nutritional supplementation to mitigate the risk of osteoporosis and fractures following bariatric surgery.

In the study by Luhrs et al. [37], which included 28 postmenopausal women undergoing either Roux-en-Y gastric bypass or sleeve gastrectomy, a notable decrease in total body BMC was observed from 2358.32 to 2280.68 g after one year, with significant reductions in BMD and BMC in the ribs and spine, regardless of the surgery type. Similarly, Ieong et al. conducted a retrospective review of 40 patients and found that both RYGB and SG led to significant decreases in BMD across multiple sites, including the femoral neck and total hip, within two years post-surgery. They also identified that reductions in serum 25-hydroxy vitamin D and calcium levels were significantly associated with decreased BMD. In a similar manner, the study by Luhrs et al. emphasized the importance of postoperative DEXA screenings for high-risk women, aligning with Ieong et al.’s conclusion that close monitoring and supplementation of vitamin D and calcium are crucial [38]. However, while Luhrs et al. [37] focused specifically on postmenopausal women with a one-year follow-up, Ieong et al. [38] included a broader patient population with a longer follow-up period and highlighted the biochemical factors influencing bone health. This comparative analysis underscores the consistent finding of bone loss following bariatric surgery and the necessity for individualized postoperative care to mitigate these adverse effects.

In a broader context, Zhao Tian et al. [39] conducted a meta-analysis that demonstrated significant alterations in the serum levels of 25-hydroxyvitamin D, calcium, phosphorus, and parathyroid hormone (PTH) following GB. Specifically, their findings indicated a decrease in vitamin D (mean difference, MD = −1.85) and calcium (MD = −0.15), alongside increases in phosphorus (MD = 0.22) and PTH (MD = 3.58) in patients post-GB. This suggests a potential worsening of bone health in these patients. In a similar manner, the study by Veeravich Jaruvongvanich et al. [40] found that patients undergoing SG experienced significant decreases in the bone mineral density (BMD) at the total hip (MD = −0.06 g/cm²) and femoral neck (MD = −0.05 g/cm²) but no significant change at the lumbar spine. Their findings also showed an increase in serum calcium and 25-hydroxyvitamin D levels post-SG, contrasting with the results seen in GB patients. These discrepancies highlight the complex nature of bariatric surgery’s impact on bone metabolism and underscore the need for tailored postoperative interventions to mitigate potential adverse effects on bone health.

From a clinical perspective, the consistent evidence of negative effects on bone health following sleeve gastrectomy in adolescents underscores the necessity for strategies to mitigate bone loss. Recommendations include optimized nutritional supplementation with essential nutrients like protein and minerals, alongside calcium and vitamin D; encouraging physical activity, specifically weight-bearing and resistance exercises to strengthen bones; considering pharmacological interventions to inhibit bone resorption or stimulate formation in high-risk individuals; regular monitoring through DXA and, if possible, HRpQCT to detect early changes; and a multidisciplinary approach involving endocrinologists, nutritionists, and physical therapists. Clinicians should discuss these potential risks and the benefits of weight loss with patients and their families to carefully consider the long-term skeletal impacts of SG.

The relationship between obesity and bone health in adolescents is inherently complex and remains a subject of ongoing debate, as studies have reported inconsistent effects on bone mineral density across different anatomical regions. The studies included in our investigation selected participants who exhibited healthy bone profiles at the onset of the study, with the purpose of minimizing confounding variables and ensuring that the baseline bone health did not skew the outcomes. Baseline assessments revealed that the BMD and Z-scores of these adolescents were within or slightly above the normal ranges for their demographic, providing a stable foundation for evaluating the effects of sleeve gastrectomy. Therefore, this initial bone mass and quality are critical, as they significantly influence post-surgical skeletal health outcomes.

### 4.2. Limitations

This review is limited by the small number of studies and participants, with data being available from nine studies involving varying sample sizes and study designs. The heterogeneity of measurement techniques, follow-up durations, and participant characteristics may affect the generalizability of the findings. The absence of studies extending beyond two years post-sleeve gastrectomy hinders our ability to understand the long-term trajectory of bone health, including whether the bone density continues to decline, stabilizes, or potentially recovers over time. Consequently, this limitation restricts the assessment of enduring risks such as osteoporosis and fractures, making it challenging to develop effective long-term management and intervention strategies for maintaining skeletal health. Additionally, differences in supplementation protocols, physical activity levels, and compliance were not thoroughly explored, which could influence bone outcomes. Only one randomized controlled trial was included, and the remaining studies were observational, limiting the ability to establish causality.

## 5. Conclusions

These findings underscore that sleeve gastrectomy in obese adolescents and young adults generally exerts a detrimental impact on bone health, despite some variability across different studies. While certain assessments revealed no significant alterations in bone mineral density in specific anatomical regions, a notable trend emerged showing substantial declines in bone density at critical sites such as the femoral neck and total hip. Additionally, the bone microarchitecture exhibited marked deterioration, characterized by a reduced trabecular number, increased trabecular separation, and thinning of cortical bone, which collectively compromise the structural integrity and strength of the skeleton. Elevated bone turnover markers further indicated an imbalance favoring bone resorption over formation, suggesting accelerated bone loss and remodeling processes post-surgery. Moreover, significant increases in marrow adipose tissue were observed, reflecting a shift in the bone marrow composition that may hinder bone formation and exacerbate bone fragility. These multifaceted changes highlight the complex and potentially adverse effects of sleeve gastrectomy on the skeletal system, emphasizing the necessity for vigilant monitoring and proactive strategies to preserve bone health in this vulnerable population.

## Figures and Tables

**Table 1:** Characteristics of included studies.

Study No.	Author(s)	Year	Country	Design	Sample Size (n)	Age Range (Years)	Quality Score (NOS)
1	Bredella et al. [20]	2017	USA	Prospective Cohort	10	14–22	7
2	Misra et al. [21]	2018	USA	Prospective Cohort	44 (22 SG, 22 NS)	14–22	8
3	Weiner et al. [22]	2020	USA	Observational Cohort	197 (99 SG, 98 LAGB)	13–20	7
4	Misra et al. [23]	2020	USA	Prospective Cohort	48 (24 SG, 24 NS)	14–22	8
5	Mitchell et al. [24]	2022	USA	Observational Cohort	66 (30 SG, 36 NS)	13–24	7
6	Järvholm et al. [25]	2023	Sweden	Randomized Controlled Trial	50 (25 SG, 25 INT)	13–16	9
7	Huber et al. [26]	2023	USA	Prospective Cohort	54 (25 SG, 29 NS)	13–24	8
8	Huber et al. [27]	2023	USA	Prospective Cohort	59 (29 SG, 30 NS)	13–25	8
9	Nimmala et al. [28]	2022	USA	Observational Cohort	64 (30 SG, 34 NS)	13–25	7

SG: sleeve gastrectomy; NS: Non-Surgical Control; LAGB: laparoscopic adjustable gastric banding; INT: Intensive Non-Surgical Treatment.

**Table 2:** Changes in areal bone mineral density following sleeve gastrectomy.

Study No.	Author(s)	BMD Site(s) Assessed for Baseline aBMD (g/cm² or Z-Score)	Baseline aBMD (g/cm² or Z-Score)	Follow-Up aBMD (g/cm² or Z-Score)	Change (%)	*p*-Value	Follow-Up
1	Bredella et al. [20]	Lumbar Spine	1.064 ± 0.241 g/cm²	0.648 ± 0.177 g/cm²	−3.7 ± 7.5%	Not significant	12 months
2	Misra et al. [21]	Femoral Neck, Total Hip	Not provided	Decrease	FN: −6.9%; TH: −4.7%	*p* = 0.0007 (FN), *p* = 0.0004 (TH)	12 months
3	Weiner et al. [22]	Not Specified	Not provided	Not provided	Not specified	Not specified	6 and 12 months
4	Misra et al. [23]	Femoral Neck, Total Hip	Not provided	Decrease	FN: −8.9%; TH: −8.4%	*p* < 0.001	12 months
5	Mitchell et al. [24]	Lumbar Spine	Not provided	No significant change	N/A	Not significant	24 months
6	Järvholm et al. [25]	Whole-Body BMD Z-score	−0.15 (−0.90, 0.43)	No significant change	N/A	Not significant	24 months
7	Huber et al. [26]	Lumbar Spine (vBMD mg/cm³)	261 ± 25 mg/cm³	245 ± 25 mg/cm³	−6%	*p* < 0.001	24 months
8	Huber et al. [27]	Lumbar Spine (vBMD mg/cm³)	261 ± 30 mg/cm³	249 ± 29 mg/cm³	−4.60%	*p* < 0.001	12 months
9	Nimmala et al. [28]	Total Hip BMD Z-score	1.50 (0.75, 2.1)	Decrease of −0.75 (−1.25, −0.40)	Significant decrease	*p* < 0.001	12 months

BMD: bone mineral density; aBMD: areal bone mineral density; FN: femoral neck; TH: total hip; vBMD: volumetric bone mineral density; *p*-value: Probability Value; N/A: Not Applicable.

**Table 3:** Changes in bone microarchitecture, assessed by HRpQCT.

Study No.	Author(s)	Bone Site	Microarchitectural Changes	Change (%)	*p*-Value
1	Bredella et al. [20]	N/A	N/A	N/A	N/A
2	Misra et al. [21]	Distal Tibia	Decrease in trabecular number; increase in trabecular separation	−8.6% (trabecular number); +10.4% (trabecular separation)	*p* = 0.004
3	Weiner et al. [22]	N/A	N/A	N/A	N/A
4	Misra et al. [23]	Hip (Narrow Neck, Intertrochanteric)	Decrease in cortical thickness; increase in buckling ratio	−0.02 ± 0.01 cm (cortical thickness); +0.80 ± 0.44 (buckling ratio)	*p* = 0.005; *p* = 0.004
5	Mitchell et al. [24]	Distal Radius, Tibia	Decrease in trabecular vBMD	Radius: −10.4%; tibia: −6.1%	*p* < 0.001
6	Järvholm et al. [25]	Spine (BMD Z-score)	Decrease in BMD Z-score	Mean difference −0.9 (−1.2 to −0.6)	Not specified
7	Huber et al. [26]	Lumbar Spine	Decrease in vertebral strength	−728 N ± 691 (−6.7%)	*p* < 0.001
8	Huber et al. [27]	Lumbar Spine	Decrease in vertebral strength	−609 N ± 838 (−5.5%)	*p* < 0.001
9	Nimmala et al. [28]	Radius and Tibia	Decrease in trabecular vBMD at radius	Radius: −7.00 mgHA/cm³	*p* = 0.002

HRpQCT: high-resolution peripheral quantitative computed tomography; BMD: bone mineral density; vBMD: volumetric bone mineral density; N/A: Not Applicable.

**Table 4:** Changes in bone turnover markers following sleeve gastrectomy.

Study No.	Author(s)	Marker	Baseline Level	Follow-up Level	Change (%)	*p*-Value
1	Bredella et al. [20]	N/A	N/A	N/A	N/A	N/A
2	Misra et al. [21]	N/A	N/A	N/A	N/A	N/A
3	Weiner et al. [22]	Osteocalcin (ng/mL)	23.66 ± 13.38	32.23 ± 13.38	33.70%	*p* < 0.001
		CTX (pg/mL)	614.37 ± 267.71	967.86 ± 267.71	57.50%	*p* < 0.001
4	Misra et al. [23]	N/A	N/A	N/A	N/A	N/A
5	Mitchell et al. [24]	P1NP (ng/mL)	95.7 ± 69.8	90.8 ± 34.4	−5.10%	Not significant
		CTX (ng/mL)	0.57 ± 0.33	0.61 ± 0.30	7.00%	*p* = 0.022
6	Järvholm et al. [25]	N/A	N/A	N/A	N/A	N/A
7	Huber et al. [26]	N/A	N/A	N/A	N/A	N/A
8	Huber et al. [27]	N/A	N/A	N/A	N/A	N/A
9	Nimmala et al. [28]	P1NP (µg/L)	78.1 (64.2, 105.9)	+9.8 (−9.9, 29.9)	Increase	*p* = 0.007
		CTX (ng/mL)	0.49 (0.37, 0.70)	+0.27 (0.10, 0.37)	Increase	*p* = 0.0001

CTX: C-Terminal Telopeptide; P1NP: Procollagen Type 1 N-Terminal Propeptide; N/A: Not Applicable.
